# Cultureless enumeration of live bacteria in urinary tract infection by single-cell Raman spectroscopy

**DOI:** 10.3389/fmicb.2023.1144607

**Published:** 2023-03-23

**Authors:** Jingkai Wang, Kang Kong, Chen Guo, Guangyao Yin, Siyu Meng, Lu Lan, Liqiang Luo, Yizhi Song

**Affiliations:** ^1^Suzhou Institute of Biomedical Engineering and Technology, Chinese Academy of Sciences, Suzhou, China; ^2^Department of Chemistry, College of Sciences, Shanghai University, Shanghai, China; ^3^Division of Life Sciences and Medicine, School of Biomedical Engineering (Suzhou), University of Science and Technology of China, Suzhou, China; ^4^VibroniX, Inc., Suzhou, China; ^5^Chongqing Guoke Medical Technology Development Co., Ltd., Chongqing, China

**Keywords:** single-cell, Raman spectroscopy, urinary tract infection, sodium acetate, live cell enumeration

## Abstract

Urinary tract infections (UTIs) are the most common outpatient infections. Obtaining the concentration of live pathogens in the sample is crucial for the treatment. Still, the enumeration depends on urine culture and plate counting, which requires days of turn-around time (TAT). Single-cell Raman spectra combined with deuterium isotope probing (Raman-DIP) has been proven to identify the metabolic-active bacteria with high accuracy but is not able to reveal the number of live pathogens due to bacteria replication during the Raman-DIP process. In this study, we established a new approach of using sodium acetate to inhibit the replication of the pathogen and applying Raman-DIP to identify the active single cells. By combining microscopic image stitching and recognition, we could further improve the efficiency of the new method. Validation of the new method on nine artificial urine samples indicated that the exact number of live pathogens obtained with Raman-DIP is consistent with plate-counting while shortening the TAT from 18 h to within 3 h, and the potential of applying Raman-DIP for pathogen enumeration in clinics is promising.

## Introduction

Urinary tract infections (UTIs) are one of the most common clinical infections worldwide. Statistics showed that about half of female adults will suffer from at least one UTI in their lifetime (Alós, 2005; Bono et al., 2022) and the overall incidence of UTIs among older adults is high (Ruben et al., 1995; Jackson et al., 2004). According to a previous study on global disease data, in 2019, UTIs has caused 404.61 million cases and 236,790 deaths (Yang et al., 2022). Therefore, UTIs have not only posed a great threat to public health but also brought a heavy burden to hospitals and communities.

For a long time, urine culture has been regarded as the gold method for UTI diagnosis in clinical settings, which includes bacterial identification and enumeration (Schmiemann et al., 2010). The enumeration illustrates the actual concentration of live bacteria in a unit urinary sample, which is a primary and direct indicator for UTI pathological diagnosis. Usually, bacteriuria, a urinary pathogen of ≥10^5^ colony-forming units (CFU) per ml, will be confirmed as a UTI (Schmiemann et al., 2010; Rowe and Juthani-Mehta, 2014). However, the current gold standard of bacterial enumeration is time-consuming due to the required overnight culturing, which generally takes more than 18 h. Such a turn-around time (TAT) delays the timely UTI diagnosis and causes empirical drug administration.

To obtain a relatively reliable quantitative estimation, imaging and molecular methods have appeared that are applicable in microbial detection. Recently, direct microscopic observation has been reported as feasible in clinical UTI diagnosis (Hiraoka et al., 1993; Beyer et al., 2019). However, the specificity and sensitivity of this method vary with the labor’s expertise in microscopy. Furthermore, direct observation is limited in reflecting the viability of the bacteria; hence, the enumeration results may include the number of pathogens unrelated to infections. In laboratory settings, fluorescent *in situ* hybridization (FISH) staining of viable cells and live–dead staining combined with microscopic enumeration have demonstrated their great potential in the enumeration (Garcia-Armisen and Servais, 2004; Kumar et al., 2011; Perdana et al., 2012). Those cell counting methods were further developed through cell sorting techniques, such as fluorescent-activated cell sorting (FACS) (Friedrich and Lenke, 2006; Doherty et al., 2010; Chen et al., 2012). In addition to the complicated procedures, self-quenching, hybridization efficiency of the probes, and reagent toxicity may lead to high-frequency false-negative counting results (Cui et al., 2012; Maslov et al., 2018), which make it impeded from clinical UTI diagnosis. Besides, nucleic acid amplification techniques, such as real-time quantitative polymerase chain reaction (RT-qPCR), are frequently used to quantify the nucleic acids extracted from the samples, widely used in environmental diagnosis and healthcare (Maurin, 2012; Bouchez et al., 2016). However, RT-qPCR detection is usually conducted with fully lysed cells instead of viable cells; thus, the quantity obtained from RT-qPCR may include metabolic-inactive cells, which introduces a false-positive result. Hence, a rapid, facile, reliable, and metabolism-sensing technique would be a thoughtful solution to improve the gold standard clinical UTI diagnosis.

Single-cell Raman spectroscopy is a label-free, non-invasive, and molecular-specific technology and has been shining a light on pathogenic microbial studies, such as microbial identification (Hu et al., 2022; Wang et al., 2023). As an alternative phenotypic-metabolic characterizing technique, single-cell Raman spectroscopy combined with deuterium isotope probing (Raman-DIP) also showed great potential in improving traditional antimicrobial susceptible tests (Song et al., 2017; Yi et al., 2021). Such a technique can obtain a rough cell enumeration and the metabolic state of the cells at the same time, which might be an ideal solution for clinical UTI diagnosis. However, deuterium labeling in the current method requires a minimum culture of 2 h (Yi et al., 2021), which indicates that bacteria will inevitably reproduce, affecting the determination of the initial bacterial concentration.

To avoid the influence of deuterium labeling on the initial bacterial concentration and establish a rapid, facile, reliable, and Raman-DIP technique for UTI diagnosis, we hereby developed a new culture medium containing sodium acetate (SA), which meets the requirements of ensuring the single-cell bacterial metabolism, meanwhile, inhibiting the division and proliferation of bacteria. The effects of the SA-containing medium on the growth and metabolism of bacteria at different growth phases were also discussed in this study. An enumeration method was established and tested with nine artificial urine samples. The feasibility of Raman spectroscopy combined with the SA medium in diagnosing UTI was also validated by the comparison with the gold standard.

## Materials and methods

### Strains and reagents

Four common UTI pathogens (Flores-Mireles et al., 2015), including two Gram-positive (*Staphylococcus aureus* ATCC 25923, *Enterococcus faecalis* ATCC 29212) and two Gram-negative strains (*Escherichia coli* ATCC 25922, *Pseudomonas aeruginosa* ATCC 27853), were used to analyze the effect of SA on bacterial growth. In addition, as the most common Gram-positive and Gram-negative species of UTI pathogens (Flores-Mireles et al., 2015), *E. coli* and *S. aureus* were also used in determining the effect of SA on single-cell metabolism. Then, we took *E. coli* to prepare artificial urine samples because it accounts for 65% of clinical UTIs. Bacteria were cultured in Luria-Bertani (LB) broth overnight in a rotary incubator at 200 rpm and 37°C until their concentrations reached 10^9^ CFU/ml.

The culture medium developed in this study was made of 50% 2× LB broth, 40% of 99.9% filtered deuterium water, anhydrous SA, and double-distilled water. Before deuterium labeling, the pH of the medium was adjusted to 7.2 with sodium dihydrogen phosphate. Unless otherwise specified, all reagents used in this investigation were purchased from Sigma Aldrich, Shanghai, China.

### Inhibition test by overnight-plate culture

The four bacterial strains from the overnight media were seeded (1:1,000) in a 10 ml culture medium containing 16-, 32-, 64-, 128-, 256-, and 512-mM SA, respectively, and cultured in the rotary incubator for 8 h. For each SA concentration, single plate-serial dilution spotting was performed at 0, 1, 2, 4, and 8 h, respectively. Five biological replicates and a serial dilution (0-, 10-, and 100-fold) were used for each SA concentration. Ten microliter of the diluted specimen was pipetted to each sample spot. After spotting, the plates were immediately transferred to a 37°C incubator and cultured overnight. Then, the number of colonies on each plate was manually enumerated and summarized to determine the inhibition effect of different SA concentrations. The same procedures were repeated for the *E. coli* obtained from their exponential phase (3 h after the lag phase) to investigate the inhibition of bacteria under different metabolic conditions.

### Deuterium labeling and Raman fingerprint acquisition

After overnight culture, *E. coli* and *S. aureus* were seeded (1:1,000) in 10 ml culture medium containing 128-, 256-, and 512-mM SA and cultured in the rotary shaker for 4 h. At least 30 single-cell Raman spectra (SCRS) for each SA concentration were collected at 0, 1, 2, and 4 h, respectively. Before each SCRS acquisition, the specimens were washed twice with centrifugation at 7,000 rpm for 3 min and resuspended with double-deionized water. Then, 2 μl of the resuspended specimens was pipetted onto an aluminum-coated slide and ready for SCRS collection after the slide was air-dried. We used a commercial model of a home-built Raman spectrometer (R300 Confocal Raman microscope, Suzhou Institute of Biomedical Engineering and Technology, China) to perform the SCRS acquisition with an integration time of 3 s, an on-surface laser power of 7 mW, and a continuous wave laser of 532 nm.

### Establishment of the enumeration method

Single-cell metabolic indices of the *E. coli* and *S. aureus* strains were obtained from their SCRS. The metabolic index is the percentage of the integrated spectral intensity of the C–D band (2,000–2,300 cm^–1^) compared with the sum of the C–D band and the C–H band (2,800–3,100 cm^–1^). Here, we define bacteria whose metabolic index difference between the sample and the negative control was more than three times the standard deviation of the negative control as metabolic-active/live bacteria.

We used the Raman microscope to perform image stitching and recognition for the sampling area on the aluminum slide to restore the initial bacterial concentration. A self-developed image-processing software completed the process of image stitching and recognition. With the spatial coordinates of all bacteria given by the image-processing software, the confocal Raman microscope could automatically collect SCRS from all bacteria. Then, the number of all bacteria with a C-D Raman band (around 2,170 cm^–1^) was subsequently obtained, which equaled the number of live bacteria in the sampling area. In the end, the initial bacterial concentration in the sample could be obtained by multiplying the number of bacteria with the C-D band by the dilution multiples.

### Validation by artificial urinary samples

After excluding risk factors such as urinary tract discomfort and antibiotic history within half a year, three groups of healthy urine samples were provided by three volunteers in our laboratory and filtered through a 0.2-μm filter membrane before being used. Then, the centrifuged overnight cultured *E. coli* were resuspended to the original concentration by the three urine samples. For each group, the resuspended specimens were separated into three artificial groups with a final bacterial concentration of 10^4^, 10^5^, and 10^6^, respectively.

To validate the consistency of the Raman-DIP-based cell counting method, all artificial samples were treated in two ways for comparison: the conventional gold-standard method versus the Raman-DIP approach. Briefly, for the gold standard (manual enumeration), procedures conclude centrifugal washing, specimen resuspension, serial-dilution spotting (0.2 μl/sampling spot), cell culture, and manual counting; for Raman-DIP, procedures were centrifugal washing, specimen resuspension, culture with SA and D_2_O, sampling, microscopic image stitching, and enumeration with the C-D Raman signal. To simplify the difficulty of cell counting, we used a series of dilution levels (10-, 100-, and 1,000-fold) for all specimens in both approaches. Then, we chose the best dilution level for the following cell enumeration. Finally, results from the two enumeration methods were summarized and analyzed.

### Data processing and analysis

In this study, unless otherwise stated, data analysis was performed using R (Version 4.2.2, R Core Team, 2022). Metabolic indices were calculated using the package reshape2 (Version 1.4.4, Wickham, 2011) and pylr (Version 1.8.8, Wickham, 2007). Figures were generated using the Origin 9 software (OriginLab Corporation, Northampton, MA, USA). All SCRS were subtracted to 450–3,200 cm^–1^ and baseline-corrected in Labspec6 (Horiba JY, Tokyo, Japan) with a 10-degree linear baseline fitting algorithm. Then, the spectra were normalized using a total area below all the peaks within the spectral range.

## Results

### Inhibition of SA on colonial reproduction

**FIGURE 1:**
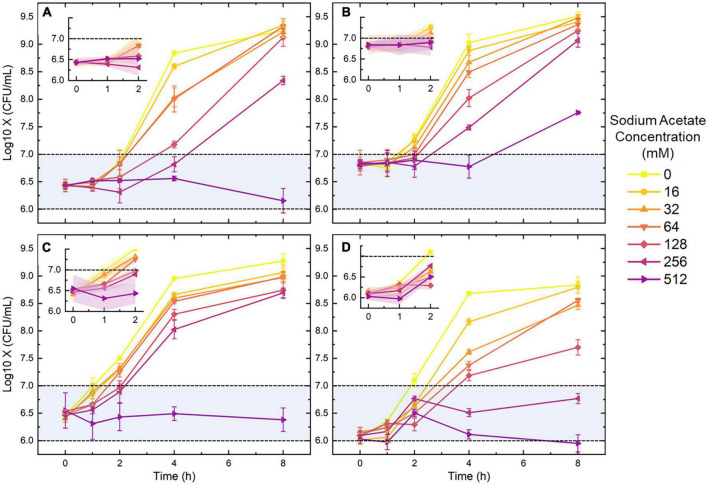
The growth curves of *Escherichia coli* **(A)**, *Staphylococcus aureus* **(B)**, *Pseudomonas aeruginosa* **(C)**, and *Enterococcus faecalis* **(D)** under the pressure of seven sodium acetate (SA) concentrations. The four detailed diagrams displaced in the upper-left corner of panels **(A–D)** are the 0-2 h growth curves of the four species. Blue regions represent the initial level of bacterial concentration. Error bars and error ribbons represent the standard deviation of five biological replicates. 0-mM SA serves as the negative control.

According to other studies, SA is an organic acid of low molecular weight, which could be excreted by bacteria and commonly used in antimicrobial and antioxidant uses (Sallam, 2007; Pinhal et al., 2019). To determine an appropriate SA concentration for growth inhibition, four bacterial strains were incubated with a series of SA concentrations. The manual enumeration results obtained from the overnight-plate culture were recorded at 5 time points (0, 1, 2, 4, and 8 h). Figure 1 demonstrates the growth curves derived from the manual enumeration results. The bacterial concentration will increase for the media containing SA lower than 128 mM as the culture time goes up; although the growth rate decreases along with the increasing SA concentration, bacteria tend to grow to a similar final concentration (around 10^8^.^75–9^.^5^ CFU/ml). On the opposite, for the medium containing 512-mM SA, the bacterial growth shows a stagnation or even reduction trend since all points of the growth curves were within the same order (the blue region). The four diagrams offered in the upper-left corner of Figures 1A–D illustrate that the bacterial growth was slow from 0 to 2 h. Especially for the SA concentration higher than 128 mM, the enumeration results are still within the same level as the initial ones. In short, the above results indicate that a medium containing 128-512 mM SA can effectively inhibit the growth and proliferation of bacteria, especially for 0-2 h.

**FIGURE 2:**
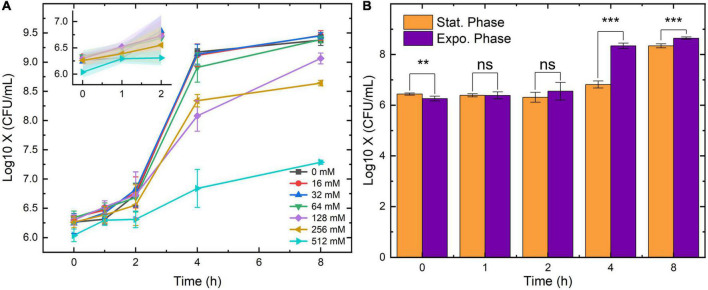
The growth curves of exponential *Escherichia coli* under the pressure of seven SA concentrations **(A)** and the growth comparison between the stationary and the exponential *E. coli* under 256-mM SA **(B)**. 0-mM SA serves as the negative control. Error bars and error ribbons represent the standard deviation of five biological replicates. Statistical significance was calculated (*t*-test) and marked accordingly. ***p ≤ 0.001; **p > 0.05, no significance (ns).

The above results were obtained from the stationary-phase bacteria which are commonly found in the LB broth after overnight culture. However, clinical UTI samples also contain bacteria in other growth phases. To investigate the inhibition of SA on bacteria obtained from a different growth phase, the growth of the exponential-phase *E. coli* was also measured in this study. The 0-8 h as well as the 0-2 h growth curves of the exponential-phase *E. coli* are displayed in Figure 2A. During 0-2 h, the number of bacteria maintained in the same colony-forming unit (CFU) level. After an eight-hour culture, the final concentration of the bacteria under 16-, 32-, and 64-mM SA came to the same point (around 10^9^.^25^ CFU/ml). Yet, the final counts of bacteria growing under 128-, 256- and 512-mM SA were lower than that under other concentrations. To visualize the growth difference under the pressure of SA, the enumeration results between the stationary and the exponential *E. coli* were compared in Figure 2B. For the comparison, we chose an SA concentration of 256 mM for its relatively mild antimicrobial characteristics and better growth-inhibition effect (Figure 1A). As predicted by Figures 1A, 2B, the concentration of *E. coli* from both growth phases showed no difference due to the inhibition of SA; on the other hand, the concentration of the exponential *E. coli* was significantly higher than that of the stationary *E. coli* after 4-h incubation. Hence, the growth and proliferation of the exponential-phase bacteria were more active under the same SA pressure.

### Inhibition of SA on single-cell metabolism of bacteria

**FIGURE 3:**
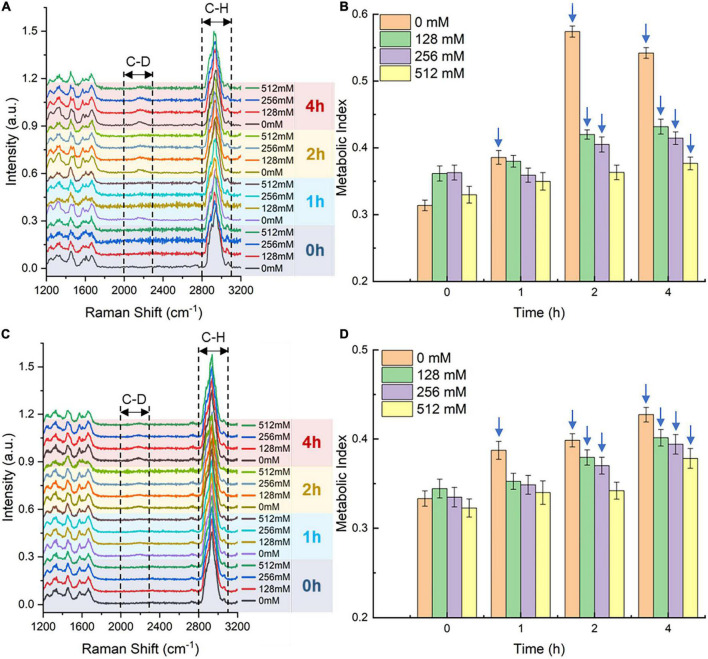
Single-cell Raman spectra (SCRS) and metabolic indices of *Escherichia coli* **(A,B)** and *Staphylococcus aureus* **(C,D)** under 128-, 256-, and 512-mM SA after different deuterium labeling times. **(A,C)** The lines represent the average intensity of at least 30 replicates. SCRS of 0-mM SA serves as the negative control. The spectra were baseline corrected and normalized using a total area below all the peaks within the spectral range. **(B,D)** Metabolic-active bacteria are annotated with blue arrows. Error bars represent the standard deviation of at least 30 replicates. The metabolic index at the 0 h time point serves as the negative control.

The Raman-DIP technique (Berry et al., 2015) was used to characterize the single-cell metabolic activity of bacteria cultured with the SA-containing medium. According to our previous study (Yi et al., 2021), metabolic-active bacteria could intake deuterium into their intracellular biomass via NADPH regeneration, and the C-H band will shift to the C-D band. Therefore, the metabolic activity of bacteria is proportional to the intensity of the C-D band compared with the sum of the C-D band and the C-H band, which was defined as the metabolic index. To determine the inhibition of SA on the single-cell metabolism indicator, the metabolic index, the *E. coli* and *S. aureus* strains were incubated with 40% D_2_O for 2 h. From the above results, we have learned that the concentration range of bacterial growth inhibition is 128-512 mM. Thus, we investigated the inhibition of SA of three concentrations (128, 256, and 512 mM) on single-cell metabolism. After 2 h of deuterium labeling, bacteria incubated with 0-, 128-, and 256-mM SA showed a notable signal of the C-D Raman band at 2170 cm^–1^ (Figures 3A, C); nevertheless, bacteria grown under the pressure of 512-mM SA presented a low C-D intensity at 2 and 4 h. To quantitatively characterize the single-cell metabolism extent, we calculated the bacterial metabolic indices under different SA concentrations and displayed the results in Figures 3B, D. Compared with the negative control (0 mM SA), the single-cell metabolism was inhibited when bacteria were incubated with an SA concentration higher than 128 mM, even though the intracellular deuterium incorporation increased with time. Meanwhile, at the same time point, the metabolic indices gradually decreased as the corresponding SA concentration increased.

In this study, bacteria whose metabolic index difference between the sample and the negative control was more than three times the standard deviation of the negative control as live bacteria. Based on that, the live bacteria were annotated with blue arrows (Figures 3B, D). Hence, a two-hour incubation time and 128 or 256 mM of SA are sufficient for deuterium labeling and activity detection of bacteria.

### Enumeration of the live bacteria

The enumeration of the live bacteria is the core of clinical UTI diagnosis. The Raman-DIP enumeration method depends on identifying single-cell bacteria with a solid C-D signal. To avoid the influence of bacterial growth on the enumeration results during deuterium labeling, a medium containing 256-mM SA was used to inhibit cell proliferation, maintain cell metabolism, and keep the bacterial concentration within the same level as in the initial specimen. The reason for selecting 256-mM SA is that the growth of bacteria is most inhibited under this concentration without affecting the metabolic activity of bacteria.

**FIGURE 4:**
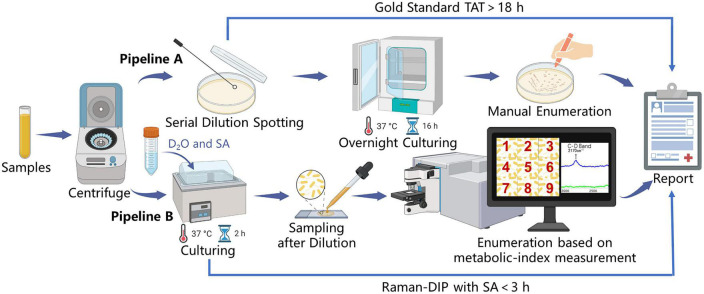
Pipelines for UTI diagnosis and comparison of the Raman-DIP method versus conventional gold-standard method. Pipeline A: manual enumeration by the conventional gold standard. Pipeline B: enumeration based on metabolic-index measurement by Raman-DIP combined with SA-containing medium.

**FIGURE 5:**
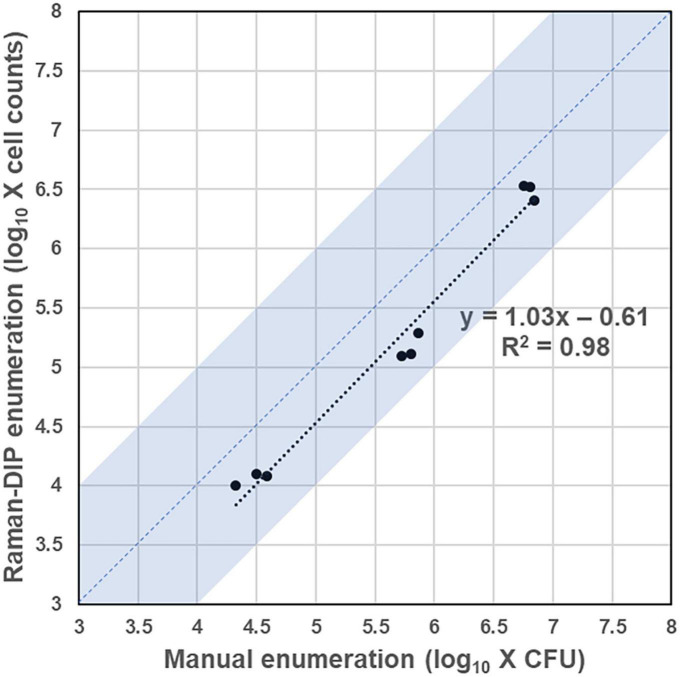
Enumeration results from the Raman-DIP method and the conventional plate-counting method. Samples with a consistent enumeration will locate in the blue region.

The enumeration method based on Raman-DIP first identifies all bacteria within the sample spot through microscopic image stitching (Supplementary Figure 1). Then, the enumeration results can be obtained by analyzing the metabolic indices of all identified bacteria, whose number equals the sum of the live bacteria. To validate the consistency of the Raman-DIP enumeration method, the conventional plate-counting procedure was also performed for comparison (Figure 4). The detailed enumeration results are shown in Supplementary Table 1. Furthermore, the enumeration results from the two methods are considered consistent if they are within the same order of magnitude (the blue region in Figure 5). Thus, samples with a consistent enumeration will locate in the blue area. From Figure 5, although bacteria cell counts are evenly 4.07 times lower than the CFU counts given by the conventional method, the nine results obtained from the two ways show positive linear correlation and 100% consistency (bacterial concentrations are within the same order of magnitude).

## Discussion

Accurate and rapid identification of the initial bacterial concentration of the specimens is necessary for the clinical UTI diagnosis. The current gold standard based on urine culture is incapable of satisfying the rapid UTI diagnosis, resulting in hidden dangers to public health. Therefore, to achieve an accurate bacterial enumeration in assisting UTI diagnosis, a modified Raman-DIP approach was developed in this study by using a culture medium to maintain the concentration of bacteria. Our study was an improvement for the Raman-DIP technique and an addition to the methods for live cell enumeration.

Although the antibacterial mechanisms are vague (Pinhal et al., 2019), previous studies have soundly proved the inhibition of SA on bacterial growth (Warnecke and Gill, 2005; De Mey et al., 2007; Carpenter and Broadbent, 2009). To inhibit cell growth while maintaining an active cell metabolism during performing Raman-DIP, we investigated the cell growth and single-cell metabolism under different SA concentrations by plate counting and assessing the metabolic indices, respectively. With the increase in SA concentration, the inhibition of bacterial growth and metabolism also intensified. Aware that bacteria in any growth phase may exist in UTI samples, we further studied the influence of SA on exponential cell growth and compared it with results from stationary cell growth. At the same SA concentration, the growth rate of bacteria in the exponential phase is higher than that in the stationary phase after 4 hours of incubation, especially for the exponential bacteria cultured under 512 mM SA, which is due to the higher metabolic activity of the exponential bacteria (Stadie et al., 2013).

To achieve a successful Raman-DIP application in UTI diagnosis, a specific SA concentration should be determined to guarantee the bacteria are metabolically active after deuterium labeling. Here, we define bacteria whose metabolic index difference between the sample and the negative control was more than three times the standard deviation of the negative control as live bacteria. The live bacteria could be identified on the above basis, and the Raman-DIP enumeration method was established (Pipeline B in Figure 4). After an inspection of the results from Figures 1, 3 and a comparison with the gold standard (Figure 5), we believe that the Raman-DIP combined with a 256 mM SA-containing medium can accurately enumerate the concentration of the live bacteria within 2 h.

Except for independent of overnight culture, a rapid counting technique is another way to decrease the TAT for clinical UTIs. For a single specimen, the Raman-DIP enumeration is based on image stitching and one-by-one metabolic index measurement. Although the samples were diluted (0-, 10-, and 100-fold) to reduce the total number of bacteria in the sampling area before Raman spectra acquisition, the enumeration still takes up to one hour. To speed up the counting procedure, stimulated Raman scattering (SRS) imaging combined with deuterium isotope probing (SRS-DIP) was tested in this study. Due to the amplification of SRS on the weak spontaneous Raman scattering, the weak C-D signal of bacteria can be enhanced by several orders of magnitude (Zhang et al., 2020). Thus, SRS can capture a Raman image within milliseconds, which would significantly shorten the time for enumeration. Supplementary Figure 2 shows the distribution of live bacteria in a part of the sampling spot, which was obtained within 10 ms. Bright pixels of the image were generated by the scattering of the intracellular C-D bond, which represented the location of the *E. coli*. However, bacterial enumeration by SRS image stitching was difficult due to the narrow imaging field and the non-stop floating of samples. To alleviate the Brownian motion of bacteria in the specimen and avoid cell damage caused by the high laser power, we attempted to turn down the laser power and perform the SRS imaging acquisition after the samples were air-dried. However, it was found hard to collect the C-D signals from the dried samples with lower laser power. In Table 1, we summarize the pros and cons of the possible cell counting solutions. Although the Raman microscopic instrument may induce a higher cost for a single cell counting test than the gold standard, the development of compact and specialized Raman spectrometers and tests of batch samples have the possibility to reduce the cost. In conclusion, our Raman-DIP with SA-containing medium offers an obvious advantage in live cell identification.

**TABLE 1:** Comparison of the current cell-counting methods.

Methods	TAT (h)	Metabolism sensing	Other problems
Gold standard	>18	Yes	/
Direct microscopy	≤1	No	Experience-dependence
RT-qPCR	2–3	No	/
Fluorescent living-cell labeling	≤2	Yes	Self-quenching, hybridization efficiency, toxicity, etc.
Raman-DIP	2–3	Yes	Cell growth during deuterium labeling
SRS-DIP with SA (This study)	2	Yes	Theoretically high efficiency for live cell counting but may cause cell damage due to high laser power
Raman-DIP with SA (This study)	2–3	Yes	/

## Conclusion

Urinary tract infections are the most common outpatient infections; acute UTIs may cause severe conditions, such as pyelonephritis and cystitis. The clinical diagnosis of UTIs is facing a critical challenge of delayed enumeration results before antibiotics administration due to the long TAT of the plate counting method. In this study, the enumeration based on Raman-DIP combined with sodium acetate-growth-inhibition has successfully identified live bacteria in urine samples and obtained accurate cell counting results within 3 h. This study demonstrated the new capability of Raman-DIP in infectious disease diagnosis other than rapid antimicrobial susceptibility test. The establishment of this proof-of-concept work can help clinicians with correct prescriptions, reduce the burden on hospitals and patients, and provide a new idea for live cell counting.

## Data availability statement

The raw data supporting the conclusions of this article will be made available by the authors, without undue reservation.

## Author contributions

JW: conceptualization, methodology, investigation, formal analysis, and writing—original draft. KK: methodology, investigation, data curation, visualization, and writing—review and editing. CG: data curation, validation, and writing—review and editing. GY: investigation and writing—review and editing. SM: software and writing—review and editing. LLan: resources and writing—review and editing. LLuo: methodology and writing—review and editing. YS: conceptualization, supervision, funding acquisition, and writing—review and editing. All authors approved the final version of the manuscript.
