# Supplementary material for: Cultureless enumeration of live bacteria in urinary tract infection by single-cell Raman spectroscopy
Source: Front Microbiol. 2023 Mar 23;14:1144607. doi: 10.3389/fmicb.2023.1144607 (PMC10076591; doi:10.3389/fmicb.2023.1144607)
Supplement: Supplementary file 1 [file Data_Sheet_1.docx]

# Cultureless enumeration of live bacteria in urinary tract infection by single-cell Raman spectroscopy

Jingkai Wang^1^, Kang Kong^1, 2^, Chen Guo^1, 3^, Guangyao Yin^1^, Siyu Meng^1^, Lu Lan^4^, Liqiang Luo^2^, and Yizhi Song^1, 3*^

^1^ Suzhou Institute of Biomedical Engineering and Technology, Chinese Academy of Sciences, Suzhou 215163, P. R. China.

^2^ Department of Chemistry, College of Sciences, Shanghai University, Shanghai 200444, P. R. China.

^3^ Division of Life Sciences and Medicine, School of Biomedical Engineering (Suzhou), University of Science and Technology of China, Suzhou 215163, P. R. China.

^4^ VibroniX, inc., Suzhou, 215123, P. R. China.

^*^ Correspondence to YZ Song ([songyz@sibet.ac.cn](mailto:songyz@sibet.ac.cn))

**Keywords:** Single cell; Raman spectroscopy; Urinary tract infection; Sodium acetate; Live cell enumeration.

## Results

### Enumeration of the metabolic-active bacteria

**Table S1.** Enumeration results of the nine artificial urine samples from the conventional method and the Raman-DIP method.

| **Sample** | **Conventional enumeration**  **(CFU/mL)** | **Raman-DIP enumeration**  **(CFU/mL)** |
| --- | --- | --- |
| Sample 1 | 10^4.5^ | 10^3.9^ |
| Sample 2 | 10^5.8^ | 10^4.9^ |
| Sample 3 | 10^6.8^ | 10^6.5^ |
| Sample 4 | 10^4.5^ | 10^3.8^ |
| Sample 5 | 10^5.8^ | 10^5.3^ |
| Sample 6 | 10^6.8^ | 10^6.4^ |
| Sample 7 | 10^4.3^ | 10^4.0^ |
| Sample 8 | 10^5.7^ | 10^5.1^ |
| Sample 9 | 10^6.7^ | 10^6.5^ |


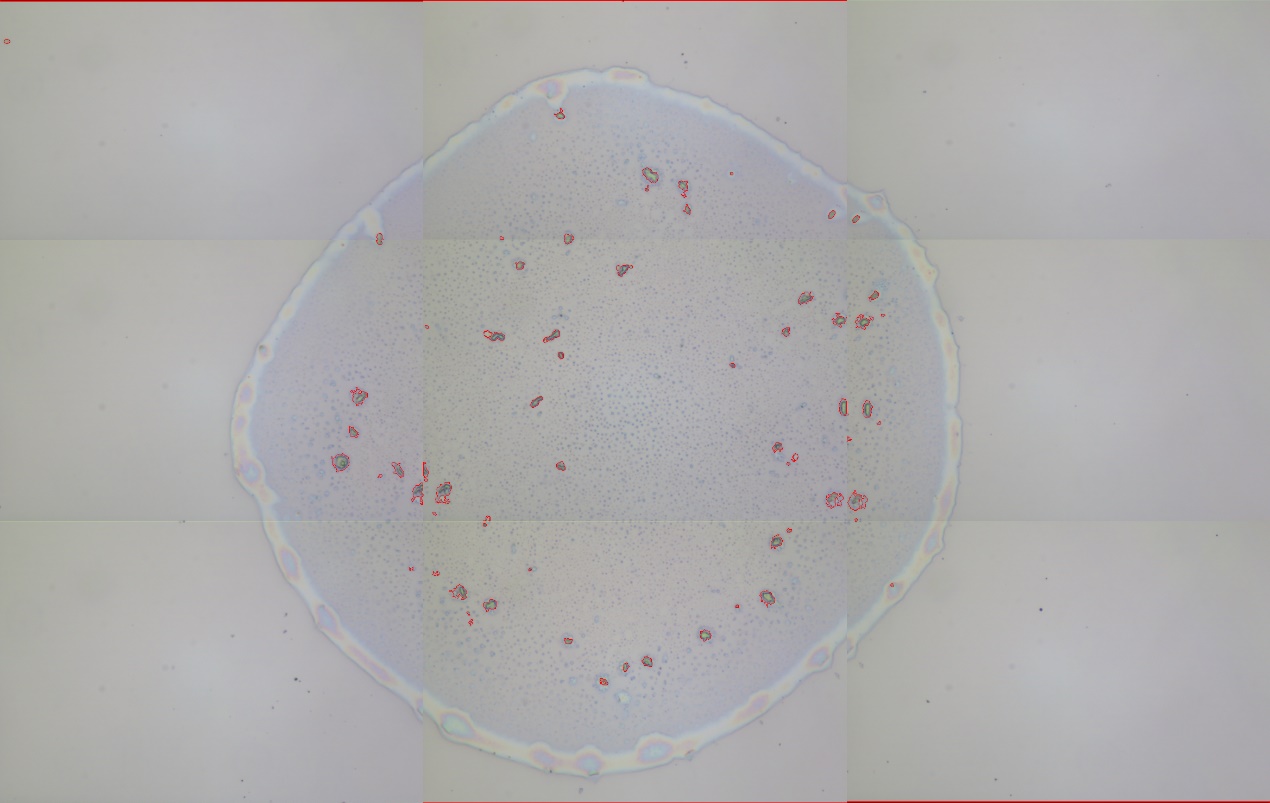


**Figure S1.** Image stitching for bacterial enumeration of the sample-spotting region. The sample spot was 0.2 μL of the overnight bacteria after twice of 1000-fold dilution. Bacteria recognized and counted by machine learning algorithm were marked with red edges.

## Discussion


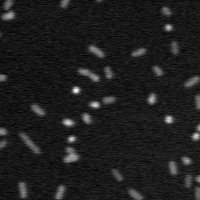


**Figure S2.** Stimulated Raman scattering imaging of live-bacteria enumeration method. Bright pixels of the image are generated by the scattering of the intracellular C-D bond. Brightness of the pixels is proportional to the intensity of the C-D signal. The image was superimposed ten times.
